# Supplementary material for: Cross-cultural adaptation and measurement properties of the Malay Shoulder Pain and Disability Index
Source: PLoS One. 2022 Mar 18;17(3):e0265198. doi: 10.1371/journal.pone.0265198 (PMC8932568; doi:10.1371/journal.pone.0265198)
Supplement: S2 Appendix — (DOCX) [file pone.0265198.s002.docx]

S2 Appendix: Malay Shoulder Pain and Disability Index (M-SPADI)
