# Supplementary figures and images for: Cross-cultural adaptation and measurement properties of the Malay Shoulder Pain and Disability Index
Source: PLoS One. 2022 Mar 18;17(3):e0265198. doi: 10.1371/journal.pone.0265198 (PMC8932568; doi:10.1371/journal.pone.0265198)

S4 Appendix. Shoulder Pain and Disability Index (SPADI) questionnaire


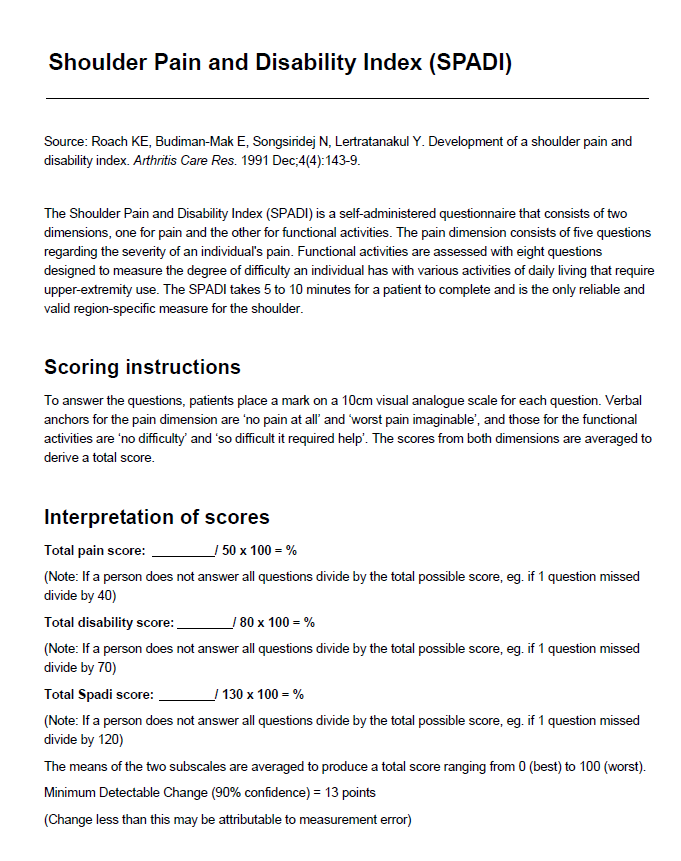


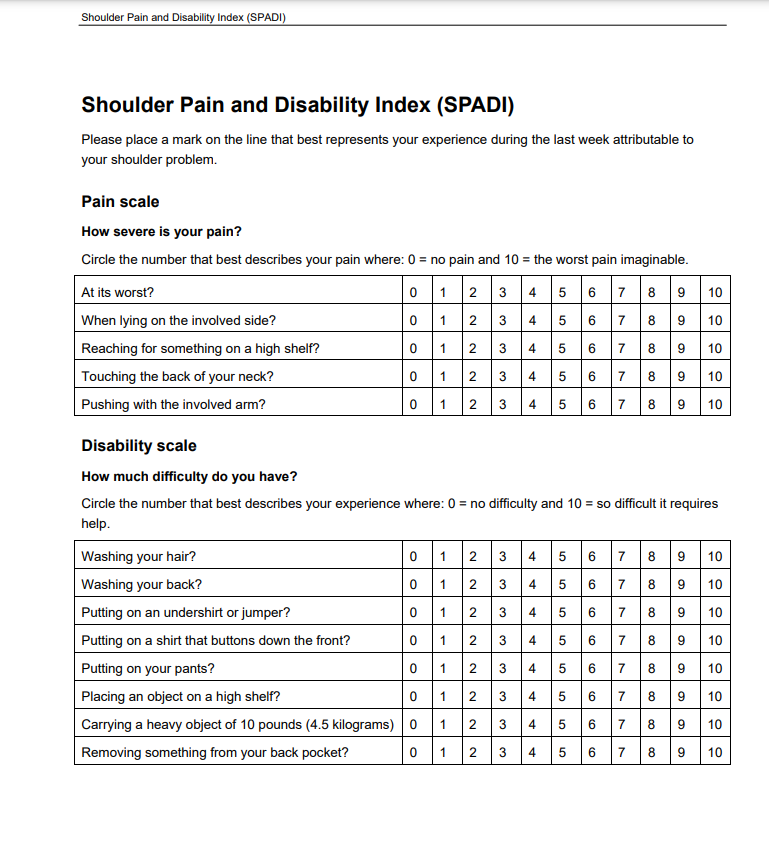

Supplement: S4 Appendix — (DOCX) [file pone.0265198.s004.docx]
