# Supplementary material for: Cross-cultural adaptation and measurement properties of the Malay Shoulder Pain and Disability Index
Source: PLoS One. 2022 Mar 18;17(3):e0265198. doi: 10.1371/journal.pone.0265198 (PMC8932568; doi:10.1371/journal.pone.0265198)
Supplement: Attachment — Submitted filename: PONE-D-21-24314_reviewer.pdf [file pone.0265198.s005.pdf]

PLOS ONE

Cross-cultural adaptation and measurement properties of the Malay Shoulder Pain and Disability Index

--Manuscript Draft--

**Manuscript Number:**	PONE-D-21-24314
**Article Type:**	Research Article
**Full Title:**	Cross-cultural adaptation and measurement properties of the Malay Shoulder Pain and Disability Index
**Short Title:**	M_SPADI Validity Study
**Corresponding Author:**	Samihah Abdul Karim, Master<br>University of Malaya Faculty of Medicine<br>Kuala Lumpur, MALAYSIA
**Keywords:**	Shoulder Pain and Disability Index; SPADI; cross-cultural adaptation; translation; measurement properties; validity; reliability
**Abstract:**	Objective :<br>The purpose of this study is to cross-culturally adapt the Malay version of the Shoulder Pain and Disability Index (M-SPADI) and evaluate its measurement properties among Malay speakers with shoulder pain.<br>Methods :<br>Cross-cultural adaptation of M-SPADI was conducted according to international guidelines. 260 participants (Shoulder pain=130, No shoulder pain=130) completed the M-SPADI, the Numerical Rating Scale (NRS), and measurement of shoulder active range of motion (AROM). 54 participants repeated M-SPADI within a mean of 9.2 days.<br>Results :<br>Cross-cultural adaptation of M-SPADI had no major issues. The M-SPADI had good face validity; item and scale content validity indexes (I-CVI, S-CVI) were >0.79 except for Disability Item 3 (I-CVI=0.75), and exploratory factor analysis showed that M-SPADI had a bidimensional structure.<br>There was a strong positive correlation between M-SPADI and NRS (r_{Pain} =0.845, $r_{Disability}$ =0.722, r_{Total} =0.795, p-value<0.001) and a negative correlation between M-SPADI and shoulder AROM with the following correlation ranges (r_{Pain} =-0.316 to -0.637, $r_{Disability}$ =-0.419 to -0.708, r_{Total} =-0404 to -0.697, p<0.001).<br>M-SPADI's total score was higher in participants with shoulder pain (Mdn: 33.8, IQR=37.3) compared to no shoulder pain (Mdn:0, IQR=0.8) and the difference was statistically significant (U=238.5, z=-13.89, p<0.001).<br>M-SPADI had no floor or ceiling effects (floor/ceiling <15%), high internal consistency (Cronbach's α_{Pain} =0.914, Cronbach's $\alpha_{Disability}$ =0.945) and good to excellent test-retest reliability (ICC_{Pain} =0.922, $ICC_{Disability}$ =0.859, ICC_{Total} =0.895).<br>Conclusion :<br>M-SPADI has a bi-dimensional structure with no floor or ceiling effects, established face, content and construct validity, internal consistency, and test-retest reliability. M-SPADI is a reliable and valid tool for assessing Malay-speaking individuals with shoulder pain in clinical and research settings.
**Order of Authors:**	Samihah Abdul Karim, Master
	Caryn-Ann Ho
	Jerri Chiu YUN LING
**Additional Information:**	
**Question**	**Response**
**Financial Disclosure**<br><br>Enter a financial disclosure statement that describes the sources of funding for the	-Samihah<br>-PV2018-62<br>-UMSC Care Fund, Faculty of Medicine University of Malaya<br>- https://umresearch.um.edu.my/funding-opportunities-forms

work included in this submission. Review the submission guidelines for detailed requirements. View published research articles from *PLOS ONE* for specific examples.<br><br>This statement is required for submission and **will appear in the published article** if the submission is accepted. Please make sure it is accurate.<br><br>**Unfunded studies**<br>Enter: *The author(s) received no specific funding for this work.*<br><br>**Funded studies**<br>Enter a statement with the following details:<br>• Initials of the authors who received each award<br>• Grant numbers awarded to each author<br>• The full name of each funder<br>• URL of each funder website<br>• Did the sponsors or funders play any role in the study design, data collection and analysis, decision to publish, or preparation of the manuscript?<br>• **NO** - Include this sentence at the end of your statement: *The funders had no role in study design, data collection and analysis, decision to publish, or preparation of the manuscript.*<br>• **YES** - Specify the role(s) played.<br><br>* typeset	- NO. The funders had no role in study design, data collection and analysis, decision to publish, or preparation of the manuscript.
**Competing Interests**<br><br>Use the instructions below to enter a competing interest statement for this submission. On behalf of all authors, disclose any competing interests that could be perceived to bias this work—acknowledging all financial support and any other relevant financial or non-financial competing interests.<br><br>This statement is required for submission and **will appear in the published article** if the submission is accepted. Please make sure it is accurate and that any funding sources listed in your Funding Information	The authors have declared that no competing interests exist.

later in the submission form are also declared in your Financial Disclosure statement.<br><br>View published research articles from *PLOS ONE* for specific examples.<br><br>**NO authors have competing interests**<br><br>Enter: *The authors have declared that no competing interests exist.*<br><br>**Authors with competing interests**<br><br>Enter competing interest details beginning with this statement:<br><br>*I have read the journal's policy and the authors of this manuscript have the following competing interests: [insert competing interests here]*<br><br>* typeset	
**Ethics Statement**<br><br>Enter an ethics statement for this submission. This statement is required if the study involved:<br><br>• Human participants<br>• Human specimens or tissue<br>• Vertebrate animals or cephalopods<br>• Vertebrate embryos or tissues<br>• Field research<br><br>Write "N/A" if the submission does not require an ethics statement.<br><br>General guidance is provided below. Consult the submission guidelines for detailed instructions. **Make sure that all information entered here is included in the Methods section of the manuscript.**	- Medical Ethics Research Committee of the University of Malaya Medical Center<br>- Approval number: MREC ID: 2020513-8617<br>- Written informed consent obtained from all participants

Powered by Editorial Manager® and ProduXion Manager® from Aries Systems Corporation

**Format for specific study types**<br><br>**Human Subject Research (involving human participants and/or tissue)**<br>• Give the name of the institutional review board or ethics committee that approved the study<br>• Include the approval number and/or a statement indicating approval of this research<br>• Indicate the form of consent obtained (written/oral) or the reason that consent was not obtained (e.g. the data were analyzed anonymously)<br><br>**Animal Research (involving vertebrate animals, embryos or tissues)**<br>• Provide the name of the Institutional Animal Care and Use Committee (IACUC) or other relevant ethics board that reviewed the study protocol, and indicate whether they approved this research or granted a formal waiver of ethical approval<br>• Include an approval number if one was obtained<br>• If the study involved *non-human primates*, add *additional details* about animal welfare and steps taken to ameliorate suffering<br>• If anesthesia, euthanasia, or any kind of animal sacrifice is part of the study, include briefly which substances and/or methods were applied<br><br>**Field Research**<br><br>Include the following details if this study involves the collection of plant, animal, or other materials from a natural setting:<br>• Field permit number<br>• Name of the institution or relevant body that granted permission	
**Data Availability**<br><br>Authors are required to make all data underlying the findings described fully available, without restriction, and from the time of publication. PLOS allows rare exceptions to address legal and ethical concerns. See the PLOS Data Policy and FAQ for detailed information.	No - some restrictions will apply

Powered by Editorial Manager® and ProduXion Manager® from Aries Systems Corporation

A Data Availability Statement describing where the data can be found is required at submission. Your answers to this question constitute the Data Availability Statement and **will be published in the article**, if accepted.<br><br>**Important:** Stating 'data available on request from the author' is not sufficient. If your data are only available upon request, select 'No' for the first question and explain your exceptional situation in the text box.<br><br>Do the authors confirm that all data underlying the findings described in their manuscript are fully available without restriction?	
**Describe where the data may be found in full sentences. If you are copying our sample text, replace any instances of XXX with the appropriate details.**<br><br>• If the data are **held or will be held in a public repository**, include URLs, accession numbers or DOIs. If this information will only be available after acceptance, indicate this by ticking the box below. For example: *All XXX files are available from the XXX database (accession number(s) XXX, XXX.).*<br>• If the data are all contained **within the manuscript and/or Supporting Information files**, enter the following: *All relevant data are within the manuscript and its Supporting Information files.*<br>• If neither of these applies but you are able to provide **details of access elsewhere**, with or without limitations, please do so. For example:<br><br>*Data cannot be shared publicly because of [XXX]. Data are available from the XXX Institutional Data Access / Ethics Committee (contact via XXX) for researchers who meet the criteria for access to confidential data.*<br><br>*The data underlying the results presented in the study are available from (include the name of the third party*	Data cannot be shared publicly because of it contains potentially identifying information of human subjects. Data are available from the RedCap UMMC Institutional Data Access / Ethics Committee (contact via email at samihahk@ummc.edu.my) for researchers who meet the criteria for access to confidential data.

and contact information or URL).<br>• This text is appropriate if the data are owned by a third party and authors do not have permission to share the data.<br><br>* typeset	
Additional data availability information:	Tick here if your circumstances are not covered by the questions above and you need the journal's help to make your data available.; Tick here if the URLs/accession numbers/DOIs will be available only after acceptance of the manuscript for publication so that we can ensure their inclusion before publication.

Powered by Editorial Manager® and ProduXion Manager® from Aries Systems Corporation

Manuscript Click here to access/download;Manuscript;M-SPADI.docx

Cross-cultural adaptation and measurement properties of the Malay Shoulder Pain and Disability Index

Caryn-Ann Ho, MBBS[1]¶, Jerri Chiu Yun Ling, MBBS, MSpMed[2]¶, Samihah Binti Abdul Karim MD, MSpMed[1*].

Affiliations:

[1]Sports Medicine Department, University Malaya Medical Centre, Kuala Lumpur, Malaysia

[2]Sports Medicine Unit, Department of Orthopaedics, Hospital Tengku Ampuan Rahimah Klang, Selangor, Malaysia

*Corrresponding author

Email: samihahk@ummc.edu.my

¶ These authors contributed equally to this work.

Abstract

Objective:

The purpose of this study is to cross-culturally adapt the Malay version of the Shoulder Pain and Disability Index (M-SPADI) and evaluate its measurement properties among Malay speakers with shoulder pain.

Methods:

Cross-cultural adaptation of M-SPADI was conducted according to international guidelines. 260 participants (Shoulder pain=130, No shoulder pain=130) completed the M-SPADI, the Numerical Rating Scale (NRS), and measurement of shoulder active range of motion (AROM). 54 participants repeated M-SPADI within a mean of 9.2 days.

Results:

Cross-cultural adaptation of M-SPADI had no major issues. The M-SPADI had good face validity; item and scale content validity indexes (I-CVI, S-CVI) were >0.79 except for Disability Item 3 (I-CVI=0.75), and exploratory factor analysis showed that M-SPADI had a bidimensional structure.

There was a strong positive correlation between M-SPADI and NRS (r_{Pain}=0.845, $r_{Disability}$=0.722, r_{Total}=0.795, p[illegible]lue<0.001) and a negative correlation between M-SPADI and shoulder AROM with the following correlation ranges (r_{Pain} =-0.316 to -0.637, $r_{Disability}$ =-0.419 to -0.708, r_{Total} =-0404 to -0.697, p<0.001).

M-SPADI's total score was higher in participants with shoulder pain (Mdn: 33.8, IQR=37.3) compared to no shoulder pain (Mdn:0, IQR=0.8) and the difference was statistically significant (U=238.5, z=-13.89, p<0.001).

M-SPADI had no floor or ceiling effects (floor/ceiling <15%), high internal consistency (Cronbach's α_{Pain}=0.914, Cronbach's $\alpha_{Disability}$=0.945) and good to excellent test-retest reliability (ICC_{Pain}=0.922, $ICC_{Disability}$=0.859, ICC_{Total}=0.895).

Conclusion:

M-SPADI has a bi-dimensional structure with no floor or ceiling effects, established face, content and construct validity, internal consistency, and test-retest reliability. M-SPADI is a reliable and valid tool for assessing Malay-speaking individuals with shoulder pain in clinical and research settings.

Introduction

Shoulder pain is a common musculoskeletal disorder with a lifetime prevalence of 7 to 67% (1, 2). Associated symptoms include restricted shoulder motion, disturbed sleep, and impaired activities of daily living (3-5). Globally, it causes work absence, disability, and increased healthcare costs (2, 6). In Malaysia, shoulder injury is ranked third in musculoskeletal disorders causing disability and fourth in the total cost of workers' compensation claims per body part (7).

Health-related patient-reported outcome measures (PROM) are essential to patient-centred care and research (8). There are at least 50 PROMs measuring shoulder function, of which the most frequently used in research include the Shoulder Pain and Disability Index (SPADI), the Constant-Murley Shoulder Scale, and the American Shoulder and Elbow Surgeons Society Standardized Shoulder Assessment Form (9). Systematic reviews concluded that no single shoulder assessment tool was superior to the other but recommended SPADI as a tool for clinical and research use (9-11).

SPADI is an English, self-administered, shoulder-specific PROM developed by Roach et al. to measure pain and disability in patients with shoulder joint disorder (1, 9). SPADI is short, easily understood, applicable in various shoulder pathology, the most responsive, and has established validity and reliability (9-11). SPADI has been cross-culturally adapted into multiple languages, including Spanish, Chinese, Thai, Marathi, Brazilian-Portuguese, Greek, Italian, Telugu, Tamil, German, Turkish, Danish, Persian, Slovene, and Dutch languages (3-5, 12-23).

There is currently no shoulder-specific PROM that has been cross-culturally adapted to the Malay language. Studies have shown that individuals with limited English proficiency find English PROMs difficult to comprehend and challenging to complete (8). In Malaysia, where

fresh graduates and students have low English proficiency (24), a Malay version of SPADI (M-SPADI) would be most beneficial as Malay is the national language and is widely spoken. Moreover, it is a compulsory language taught in all primary and secondary schools (25).

Given SPADI's high clinical value and increased use in multicultural research, M-SPADI is needed for use in the Malay-speaking population. Our objective was to cross-culturally adapt a Malay version of SPADI and evaluate its measurement properties which include face validity, content validity, structural validity, hypothesis testing for construct validity, known-group validity, floor and ceiling effects, internal consistency, and test-retest reliability. We reported this study following the COSMIN 2021 reporting guidelines (26).

Materials and methods

Study design

This study was a two-phase cross-cultural adaptation and validation of M-SPADI following standard guidelines (27-29). We received permission to cross-culturally adapt the Original SPADI to M-SPADI from Professor Roach and obtained study approval from the Medical Ethics Research Committee, University of Malaya Medical Centre (UMMC) (MREC ID: 2020513-8617). We conducted the study per the Declaration of Helsinki, and all participants gave written informed consent.

Participants

We performed universal sampling where all eligible patients attending the Sports Medicine Clinic, UMMC, were invited to join this study from 1st June 2020 to 12th May 2021. The inclusion criteria were patients attending Sports Medicine Clinic, ≥ 18 years old, and

understood the Malay language. Exclusion criteria were neck, elbow, wrist, or hand injury, decline to give consent, and psychiatric illness.

We calculated a sample size of 50 participants for the pilot study and 60 participants for test-retest (27, 30). Based on a participant to item ratio of 10: 1, the validation study sample size was 130 participants (28, 30). To assess known group validity, another 130 participants with no shoulder pain were recruited (28, 30).

Instruments

Shoulder Pain and Disability Index

This study used the numerical rating scale version of SPADI. SPADI has 13 items subdivided into two subscales which measure pain (five items) and disability (eight items) (1). The initial visual analog scale (VAS) was replaced by an 11-point numerical scale where the individual scored their level of pain or difficulty from 0 to 10 with the anchors 'no pain/ no difficulty' and ' worst pain imaginable/ so difficult it requires help' (31). Based on COSMIN guidelines and Coltman et al., the author considered SPADI a reflective construct (31, 32).

Each subscale score is calculated using the formula:

$(sum\ of\ subscales's\ item\ score \div subscale's\ maximal\ possible\ score) \times 100$ (1, 9).

The maximal possible score excludes any unmarked item but requires at least 3/5 pain items and 6/8 disability items answered for SPADI to be scored (10). The total SPADI score is the unweighted mean of pain and disability domain scores (9). The scores range from 0= the best to 100= the worst with no cut-off point to indicate severity as it was designed to measure current status and change over time (1, 9).

The Numerical Rating Scale (NRS)

The NRS is an instrument for pain intensity assessment where individuals are asked to select a number from 0 to 10 that best describes their pain intensity (33). The anchors are zero for no pain and ten for the worst pain ever possible (33).

Phase 1: Cross-Cultural Adaptation

The cross-cultural adaptation process consisted of translating SPADI from English to Malay and culturally adapting M-SPADI to the Malaysian culture (27, 34).

Stage 1: Initial translation

Three independent bilingual Malay native-speaking translators forward translated SPADI from English to Malay, producing FT1, FT2, and FT3. The naive translator was a Malay Language teacher, whereas the informed translators were an Associate Professor of Sports Medicine and a Sports Medicine Physician (27).

Stage 2: Synthesis of the translation

Two researchers synthesized FT1, FT2, and FT3 to produce FT4. Resolution of issues was by consensus, and the principal researcher acted as observer and scribe.

Stage 3: Back translation

Three independent translators back-translated FT4 from Malay to English producing BT1, BT2, and BT3. All translators were bilingual, had no medical background, were unfamiliar with the concepts explored in SPADI, and had no access to the original SPADI. The translators were an English lecturer and two English teachers.

Stage 4: Expert committee

A bilingual expert committee reviewed all the abovementioned versions of the questionnaire and, through consensus, consolidated the prefinal version of the M-SPADI. The expert panel consisted of a senior lecturer from the University Malaya Faculty of Languages and Linguistics, a Professor of Orthopaedic Surgery, a Professor of Family Medicine, two Sports Medicine Physicians, a Rehabilitation Medicine Physician, a Senior Physiotherapist, an Occupational Therapist, and an Exercise Physiologist.

Stage 5: Pilot testing of prefinal version

Fifty participants completed the prefinal version of M-SPADI and gave feedback regarding what they understood about the questions, any difficulty understanding the questions, and any suggestions for improvement. These participants were retained for the forthcoming validation study.

Stage 6: Submission of documentation to developers.

We submitted all documentation and a report of the adaptation process to the original author.

Phase Ⅱ: Validation Study (Measurement property assessment)

130 participants with shoulder pain and another 130 participants without shoulder pain filled in a written consent form and the Research Electronic Data Capture (REDCap) online survey. The survey consisted of the Demographics form (S1_Fig), the M-SPADI (S2_Fig), and the NRS. A researcher measured the active range of motion (AROM) of the affected shoulder using standard shoulder goniometer technique (35).

The first 60 participants who were not undergoing invasive procedures or starting new treatments in the week preceding and following their study enrolment date were asked to repeat

an identical M-SPADI seven days later. This was to ensure that the participant's condition was stable during testing. 56 participants out of 60 (93%) returned the second M-SPADI.

Data Management

Study data were collected and managed using the REDCap electronic data capture tools hosted by the University of Malaya. REDCap is a secure, web-based software platform designed to support data capture for research studies (36). It is compliant with the Health Insurance Portability and Accountability act (37). Strategies used to minimize missing data included collecting only essential information and designing a user-friendly online survey that can only proceed to the next page upon completing all items. Any survey which did not fulfil the SPADI scoring criteria was excluded from the study.

Statistical Analysis

Statistical evaluation of measurement properties was carried out using the IBM SPSS Statistics for Windows, version 22 (IBM Corp., Armonk, NY, USA). An α-level of 0.05 was used for all statistical tests as the significance cut-off point, and normality for all data was assessed using Q-Q plots and the Shapiro-Wilk test. Non-parametric tests were performed when data were not normally distributed. All statistical analysis result classification keys were summarised in their respective tables.

Validity

Content validity was evaluated by a panel of eight expert committee members using Content validity forms (S3_Fig). We subsequently calculated the content validity index for each item (I-CVI) and the overall scale (S-CVI) (38).

The M-SPADI's face validity was tested by assessing feedback regarding relevance and comprehensibility from 50 participants in the pilot study.

Structural validity was assessed by performing exploratory factor analysis (EFA) to determine the factor structure of M-SPADI (28). The extraction method was principal component analysis with varimax rotation and Kaiser normalization. Factors with eigenvalues ≥1 were extracted, and factor loading ≥0.5 was considered significant(39).

As there is no gold standard shoulder-specific questionnaire in the Malay language, criterion validity could not be tested (28). Hypotheses testing for construct validity was assessed using convergent validity and known-group validity (28, 29). Spearman's correlation was used to calculate the correlation between M-SPADI and the NRS and between M-SPADI and shoulder AROM. We hypothesized that there would be a strong positive correlation between the M-SPADI and the NRS. We also hypothesized that M-SPADI would have a negative correlation to shoulder AROM.

Mann-Whitney U test was performed to assess known-group validity by comparing rank means of M-SPADI scores for the shoulder pain and no-shoulder pain groups. We hypothesized that M-SPADI scores for the shoulder pain group would be higher than the no shoulder pain group, and the difference would be statistically significant.

Floor and ceiling effects were assessed and considered present if ≥15% of participants achieved the lowest or highest possible subscale or total scores (40).

Reliability

We evaluated internal consistency by calculating Cronbach α for each M-SPADI unidimensional scale (26).

Test-retest reliability was assessed using the intraclass correlation coefficient (ICC) with a 95% confidence interval based on average measurement, absolute agreement, 2-way mixed-effects model (29, 41, 42). The selected interval between repeated measures was seven days to prevent recall but ensure no clinical change had occurred (29).

RESULTS

Description of Sample

Fifty participants enrolled in the pilot study. The phase II validation study had 260 participants, consisting of the shoulder pain group (n=130) and no shoulder pain group (n=130). Participants' descriptive statistics were reported as mean and standard deviation (Table 1). The shoulder pain diagnoses were rotator cuff injuries with or without impingement (54.6%), frozen shoulder (14.6%), labral injuries (3.1%), acromioclavicular joint disease (3.8%), and others (23.9%). The median time to complete M-SPADI was 2 minutes (range 1-5 minutes), comparable to previous studies (9). Due to the online survey design, there was no missing data except for test-retest, where four participants did not complete the second M-SPADI.

Table 1: Participant demographic characteristics and descriptive statistics of M-SPADI, NRS, and shoulder active range of motion.

		Validation Study	
	Pilot Study (n=50)	Shoulder pain group (n=130)	No shoulder pain group (n=130)
Age, years (mean, SD)	46 (16.3)	52 (15.1)	42 (15.7)
Male	30(56.6%)	63(48.5%)	65(50%)
Female	23(43.4%)	67 (51.5%)	65(50%)
**Occupation**			
Employed	40 (75.4%)	75 (57.7%)	101(77.7%)
Retired	10 (18.9%)	34 (26.2%)	15(11.5%)
Homemaker	2 (3.8%)	15(11.5%)	6(4.6%)
Other	1 (1.9%)	6(4.6%)	8(6.2%)
**Level of education**			
No formal education	1 (1.9%)	1(0.7%)	0 (0%)

Primary education	1 (1.9%)	3(2.3%)	2(1.5%)
Secondary education	11 (20.6%)	40(30.8%)	23(17.7%)
Tertiary education	40 (75.5%)	86(66.2%)	105(80.8%)
**Affected Shoulder**			
Dominant	-	97(74.6%)	-
Non-Dominant	-	33(25.4%)	-
**Stage**			
Acute (<6 weeks)	-	11 (8.5%)	-
Subacute (6-12 weeks)	-	20 (5.4%)	-
Chronic (>12 weeks)	-	99 (76.1%)	-
**M-SPADI (mean, SD)**			
Pain	24.22(26.52)	44.22(24.01)	1.75(5.78)
Disability	17.54(23.77)	32.05(23.24)	1.21(3.57)
Total Score	20.11(24.21)	36.71(22.46)	1.41(4.27)
**NRS (mean, SD)**	2.95(3.18)	5.03(2.53)	0.21(0.823)
**Shoulder Active Range of Motion (mean, SD)**			
Forward Flexion	164.17(25.04)	143.95(35.01)	178.16(4.62)
Abduction	155.15 (40.4)	134.61(41.55)	178.15(4.98)
Extension	51.90(10.89)	52.68(9.242)	59.38(7.55)
Internal Rotation	50.33(26.36)	39.58(26.338)	70.81(13.06)
External Rotation	72.01(29.75)	44.35(29.92)	86.98(9.99)

M-SPADI: Malay Shoulder Pain and Disability Index, NRS: Numerical Rating Scale

Cross-Cultural Adaptation

There were no significant problems encountered in the process of cross-cultural adaptation of SPADI into Malay. Minor issues encountered during translation were due to the selection of synonyms for example, carrying→*mengangkat* or *memikul*. Several items required consensus from the expert committee, such as Pain Item 5 pushing with the involved arm →*menolak dengan lengan yang terlibat*. The sentence was confusing as one does not push with *lengan,* which means forearm. The item was then rephrased to *menolak dengan tangan yang sakit*. The term jumper in Disability Item 3 was correctly translated to *baju sejuk* but was questioned on its suitability for the Malaysian population. The expert committee decided to keep the term

baju sejuk in the pilot study. Analysis of the pilot study revealed no issues faced by participants with the term *baju sejuk*.

Validity

Content Validity

The S-CVI and I-CVI for all items were >0.79 except for Disability Item 3, which had an I-CVI=0.75 requiring amendment (Table 2). This item was "putting on an undershirt or jumper," which was amended during expert committee review. Post amendment and after pilot study outcome, the expert committee agreed to keep the item.

Table 2: Content Validity of M-SPADI

ITEM	I-CVI: Consistency	I-CVI: Representative	I-CVI: Relevance	I-CVI: Comprehensibility
Pain subscale item 1	1	1	1	1
Pain subscale item 2	1	1	1	1
Pain subscale item 3	1	1	1	1
Pain subscale item 4	1	1	1	0.87
Pain subscale item 5	1	1	1	0.87
S-CVI (Pain)	1	1	1	0.95
Disability subscale item 1	1	1	1	1
Disability subscale item 2	1	1	1	0.87
Disability subscale item 3	1	1	1	0.75
Disability subscale item 4	1	1	1	0.87
Disability subscale item 5	1	1	1	1
Disability subscale item 6	1	1	1	1
Disability subscale item 7	1	1	1	1
Disability subscale item 8	1	1	1	1
S-CVI (Disability)	1	1	1	0.83
S-CVI (Total score)	1	1	1	0.94

M-SPADI: Malay Shoulder Pain and Disability Index
I-CVI: item content validity index, S-CVI: scale content validity index
I-CVI>0.79 (appropriate), I-CVI=0.70-0.79 (requires revision), and I-CVI<0.70 (eliminated) (38)

Face Validity

The overall pilot study feedback was good, with minimal issues in understanding the M-SPADI items by participants. All participants agreed that the questionnaire was clear and easy to understand, and 86.8% gave positive feedback regarding item relevance.

Structural Validity

EFA was performed using principal component analysis with varimax rotation. The Kaiser-Meyer Olkin=0.930 and Bartlett's test of sphericity were significant ($c^2_{(78)}$ =1613.77, p <0.001). Two factors were extracted with eigenvalues >1 where factor 1 explained 66.81% of variance and factor 2 explained 9.04% of variance. Items that loaded onto factor 1 were Disability Items 1, 2, 4, 5, and 8, while items that loaded onto factor 2 were Pain Items 1, 2, 3, 5, and Disability Item 7. Pain Item 4, Disability Item 3, and 6 showed cross-loading, as demonstrated in Table 3.

Table 3: Exploratory factor analysis of M-SPADI

	Factor	
	1 (Disability)	2 (Pain)
Pain subscale item 1		0.833
Pain subscale item 2		0.834
Pain subscale item 3		0.756
Pain subscale item5		0.765
Disability subscale item 7		0.659
Disability subscale item 1	0.790	

Disability subscale item 2	0.688	
Disability subscale item 4	0.855	
Disability subscale item 5	0.857	
Disability subscale item 8	0.819	
Pain subscale item 4	0.596	0.635
Disability subscale item 3	0.738	0.506
Disability subscale item 6	0.656	0.609
Eigenvalues	8.686	1.175
Percentage of Variance	66.813%	9.039%

Extraction method: Principal component analysis

Rotation Method: varimax with Kaiser normalization

Based on a sample size of 130, significant factor loading is ≥ 0.50(39)

Underlined factor loading indicates the highest loading factor in cases of cross-loading

Hypotheses testing for Construct Validity

1. *Convergent validity*

Spearman's correlation revealed a statistically significant, strong, and positive correlation between the NRS and M-SPADI pain subscale (r=0.845, p<0.001), M-SPADI disability subscale (r=0.722, p<0.001), and M-SPADI total scores (r=0.795, p<0.001)(43). The results supported our hypothesis that M-SPADI subscales and total scores are strongly and positively correlated with the NRS.

M-SPADI pain subscale (correlation range r=-0.316 to -0.637, p<0.001), disability subscale (correlation range r=-0.419 to -0.708, p<0.001), and total scores (correlation range r=-0404 to -0.697, p<0.001) showed statistically significant negative correlation to shoulder AROM, of which majority showed moderate correlation strength confirming our hypothesis. Forward flexion had the highest overall correlation with M-SPADI subscales and the total score

(correlation range r=-0.637 to -0.708, p<0.001), while extension had the lowest correlation with M-SPADI subscales and the total score (correlation range r=-0.316 to -0.419, p<0.001) (Table 4).

Table 4: Spearman's correlation coefficient between M-SPADI, NRS, and shoulder active range of motion

	Spearman's correlation (r)		
	M-SPADI Pain	M-SPADI Disability	M-SPADI Total Score
NRS	0.845[1]	0.722[1]	0.795[1]
Forward Flexion	-0.637[1]	-0.708[1]	-0.697[1]
Abduction	-0.591[1]	-0.706[1]	-0.679[1]
Extension	-0.316[1]	-0.419[1]	-0.404[1]
Internal Rotation	-0.498[1]	-0.656[1]	-0.618[1]
External Rotation	-0.457[1]	-0.574[1]	-0.550[1]

Internal and external rotation was measured at 90° abduction, [1]p-value<0.001 M-SPADI: Malay Shoulder Pain and Disability Index, NRS: Numerical Rating Scale

Spearman's correlation strength: r=1(perfect), r=0.7-0.99 (strong), r=0.4-0.69 (moderate) r=0.1-0.39 (weak) and r=0- 0.09 (no correlation) (43)

2. Known group validity

The M-SPADI total scores were higher in participants with shoulder pain (Mdn: 33.8, IQR=37.3) compared to no shoulder pain (Mdn :0, IQR=0.80), (U=238.5, z=-13.9, p<0.001, r=0.862).

Overall Mann-Whitney U test revealed that M-SPADI pain scores, disability score, and total score were significantly higher in the shoulder pain group compared to the no shoulder pain group and the difference was statistically significant with a large effect size (Table 5).

Table 5: Mann-Whitney U results comparing participants with and without shoulder pain

M-SPADI	Group	Mean	SD	min-max	Median	IQR	Mann-Whitney U	Z score	p-value	Effect size (r)
Pain	Shoulder pain (n=130)	44.22	24.01	4-98	46.0	40.5	224.5	-14.07	<0.001	0.871
	No shoulder pain (n=130)	1.75	5.78	0-48	0	0				
Disability	Shoulder pain (n=130)	32.05	23.24	0-100	26.3	37.8	499.5	-13.60	<0.001	0.843
	No shoulder pain (n=130)	1.21	3.57	0-22	0	0				
Total Score	Shoulder pain (n=130)	36.71	22.46	2-87	33.8	37.3	238.5	-13.9	<0.001	0.862
	No shoulder pain (n=130)	1.41	4.27	0-32	0	0.8				

M-SPADI: Malay Shoulder Pain and Disability Index, SD: Standard Deviation, IQR: Interquartile range
Effect size: r≥0.20 (small), r≥0.50 (medium), r≥0.80 (large) (43, 46)

Floor and Ceiling effect

Less than 15% of participants with shoulder pain achieved the lowest or highest possible scores in the pain subscale (floor=0%, ceiling=0%), disability subscale (floor=1.5%, ceiling=0.8%), or total score (floor=0%, ceiling=0%) (Table 6).

Reliability

Internal Consistency

The M-SPADI pain and disability subscales had a high internal consistency with Cronbach's α=0.914 and 0.945, respectively (Table 6).

Table 6: Floor and ceiling values, internal consistency, and test-retest reliability of M-SPADI

				Intraclass Correlation Coefficient		
M-SPADI	Min (Floor value%)	Max (Ceiling value%)	Cronbach's α	ICC	95% CI	P-value
Pain	4 (0%)	98(0%)	0.914	0.922	0.867-0.954	<0.001
Disability	0 (1.5%)	100 (0.8%)	0.945	0.859	0.759-0.917	<0.001
Total Score	1.5 (0%)	86.9 (0%)	-	0.895	0.821-0.938	<0.001

ICC: Intraclass correlation coefficient, CI: confidence interval
Floor value % and ceiling value %: frequencies of floor and ceiling values in percentage
Cronbach α=0.70-0.95 is good internal consistency, Cronbach α ≥0.95 redundancy in items(29, 41)
ICC<0.500 poor, ICC=0.500-0.749 moderate, ICC=0.750-0.900 good, ICC >0.900 excellent (42).

Test-retest Reliability

Fifty-six participants completed the test-retest with a mean time of 9.2±3.8 days between the first and second tests. The ICC results of the M-SPADI pain subscale, disability subscale, and total scores (ICC_{Pain}=0.922, $ICC_{Disability}$=0.859, ICC_{Total}=0.895, p<0.001) revealed good to excellent test-retest reliability (42).

Discussion

The cross-cultural adaptation of M-SPADI adhered strictly to recommended guidelines (28, 29). During the expert committee review, there were two main issues. The first was Disability Item 3: "putting on an undershirt or jumper," which scored I-CVI=0.75. This item had issues with the word undershirt and jumper, thus requiring multiple amendments and a trial in the pilot study before being accepted by the expert committee. This was a similar issue faced by the Brazilian-Portuguese study, which substituted the word "jumper" for "T-shirt" followed by the "term over your head" (13). The pilot study results showed no participant had issue with the item *memakai singlet atau baju sejuk*, and the subsequent decision by the expert committee

and researchers was to accept the item. The second issue was ensuring M-SPADI was accessible and generalizable to all Malay-speaking individuals from various education and geographic backgrounds. This was accomplished by following the recommendation that PROMs should be simple enough for a 12- year old to understand (27). Some initial Malay terms were accurate translations but were complicated and not commonly used. Simpler Malay words were selected; for example, *tengkuk* was replaced with *belakang leher*, and *mengenakan* was changed to *memakai*. Based on the pilot study feedback and the Content Validity Index, M-SPADI has good face validity and content validity.

EFA showed that M-SPADI has a bi-dimensional structure with five items loading onto factor 1 and another five items loading onto factor 2. The three items which showed cross-loading were Pain Item 4, Disability Item 3, and Disability Item 6, all of which were overhead activities. Even though these items showed cross-loadings, they all had higher loadings on their predicted factors. The M-SPADI EFA revealed that items involving overhead activities tended to load for both pain and disability, while items regarding carrying heavy objects tend to load with pain despite being categorized as a disability item. Our EFA findings were similar to MacDermid et al. and may suggest that respondents cannot distinguish between pain and disability in some functional items as these two factors are closely related (44, 45).

Other studies which had similar EFA results of two factors with some items cross-loading were the original SPADI (1), Spanish SPADI (3), and Slovene SPADI (22). The Chinese SPADI reported two distinct factors with no cross-loading (4), while other studies reported a single factor (23, 45) and up to 3 factors (19).

Convergent validity was established by comparing M-SPADI to the NRS and comparing M-SPADI to shoulder AROM. M-SPADI had a stronger positive correlation with the NRS when compared to the Spanish and Chinese SPADI studies (3, 4). These studies both compared their

respective SPADI scores to the VAS pain score, which resulted in moderate correlation for the pain subscale ($r_{Chinese/Spanish}$=0.488/0.670), weak to moderate correlation strength for the disability subscale($r_{Chinese/Spanish}$=0.313/0.650), and moderate correlation for total scores($r_{Chinese}$=0.402) (3, 4). Differing correlation strengths could be due to the studies utilising different pain scales which were the NRS and the VAS (3, 4).

Spearman's correlation proved the hypothesis that M-SPADI subscales and total scores were negatively correlated with shoulder AROM. Previous studies which performed similar analyses include the Original SPADI (1), Telugu SPADI (16), and Tamil SPADI (17). These studies reported a negative correlation between SPADI scores and shoulder AROM (1, 16, 17).

While all four abovementioned studies reported a negative correlation between SPADI scores and shoulder AROM, they reported different findings when comparing which scale had the highest and lowest correlation with shoulder AROM. M-SPADI disability subscale had the best correlation with AROM, whereas Telugu and Tamil SPADI pain subscale had the best correlation with AROM (r_{Malay}= -0.416 to -0.708, r_{Telugu}=-0.403 to -0.536, r_{Tamil}=-0.455 to -0.585) (16, 17). Regarding the lowest correlation, M-SPADI pain subscale, Telugu SPADI total score, and Tamil SPADI disability subscale scored the lowest correlation with AROM (r_{Malay}= -0.316 to -0.637, r_{Telugu}=-0.350 to -0.505, r_{Tamil}=-0.482 to -0.588) (16, 17). Original SPADI reported similar results for all three scales with moderate to strong correlation strength ($r_{original}$=-0.520 to -0.803) (1). Differences in the findings between M-SPADI, Tamil SPADI, and Telugu SPADI studies could be due to the differences in the severity and duration of shoulder disease at the time of data collection as the mean of the total score for M-SPADI was much lower compared to Telugu and Tamil ($Mean_{Malay}$:36.71, $Mean_{Telugu}$:81.31, $Mean_{Tamil}$:52.60) (16, 17). Mean scores of AROM were also much higher in the M-SPADI

study than the Telugu SPADI and Tamil SPADI study (Example: $Abduction_{Malay}$=134.61°, $Abduction_{Telugu}$=83.00°, $Abduction_{Tamil}$ =103°) (16, 17).

Regarding shoulder AROM, Original SPADI and Telugu SPADI had similar results where extension had the highest correlation with SPADI scales ($r_{Original}$=-0.769 to -0.803, r_{Telugu}=-0.386 to-0.536) (1, 16). This differed from M-SPADI and Tamil SPADI, which reported forward flexion as having the highest correlation with SPADI scores (r_{Malay}=-0.637 to -0.708, r_{Tamil}=-0.577 to -0.588) (17). A possible factor for these differing findings is the effect of different diagnoses on shoulder AROM. Most M-SPADI participants had rotator cuff injuries with or without impingement which we predict to have the most decrease in forward flexion and abduction and the least effect on extension. Comparatively, with diseases such as frozen shoulder, we expect a global reduction of range of motion.

Known-group validity testing demonstrated that the M-SPADI subscale and total scores were higher in the group with shoulder pain compared to the group without shoulder pain, and the difference was statistically significant. This confirmed that M-SPADI could discriminate between different groups, in this case, participants with and without shoulder pain. Other studies with reported known-group validity for SPADI include Dutch SPADI, Slovene SPADI, and Danish SPADI. They compared high and low initial pain scores (23), work absence versus no work absence (23), different severities of self-reported perceived disability (22), and working versus non-working participants (20).

Cronbach α for total score was not calculated as our EFA concluded that M-SPADI is bidimensional (26, 28). Instead, we reported Cronbach α for each unidimensional domain: the pain subscale (Cronbach's α=0.914) and the disability subscale (Cronbach's α=0.945). These results are comparable to those reported in the original SPADI and other SPADI translations

(1, 4, 13, 14, 16, 18). These results show that the M-SPADI has a high internal consistency and does not have item redundancy as it did not cross the Cronbach's $\alpha>0.95$ threshold.

The test-retest reliability for M-SPADI was rated good to excellent ($ICC_{Pain}=0.922$, $ICC_{Disability}=0.859$, $ICC_{Total}=0.895$) and was superior to the original SPADI, which had moderate test-retest reliability ($ICC_{Pain}=0.655$, $ICC_{Disability}=0.644$, $ICC_{Total}=0.655$) (1). The Original SPADI findings could be due to its small sample size of 37 males. A recent systematic review reports test-retest reliability of SPADI ranges from ICC=0.850-0.922, reflecting the findings of this study (9).

The strengths of this study were its large sample size fulfilling the COSMIN guidelines requirements (26, 28). Moreover, this study adhered to the standard methods and guidelines for all procedures. In addition, this study includes known-group validity comparing participants with and without shoulder pain which has not been performed before.

The limitation of this study was that it was conducted in a single urban centre, with 75% of participants having tertiary education, which may cause bias in the results. A multicentre study conducted in rural and urban settings with participants of different educational backgrounds could yield different results. We also noted that the mean time interval for the test-retest was 9.2 ± 3.8 days, which was longer than the recommended seven days (9). Like the Danish SPADI study, the M-SPADI results did not seem to be affected by this prolonged interval, and the results were in keeping with other SPADI translation studies (13, 14, 16-18, 20-22).

We recommend future prospective studies for the M-SPADI to examine measurement invariance, measurement error, and responsiveness in multicentre studies involving rural and urban populations.

Conclusion

The M-SPADI has a bi-dimensional structure with good face and content validity, established construct validity, good internal consistency, and good to excellent test-retest reliability. It also has no floor or ceiling effect. Overall, the M-SPADI is a reliable and valid tool to assess pain and disability in Malay-speaking individuals with shoulder pain in clinical and research settings.

Acknowledgement

The authors would like to thank Associate Professor Dr. Mohamad Shariff Bin A Hamid (University of Malaya) for his guidance throughout this study.

References

1. Roach KE, Budiman-Mak E, Songsiridej N, Lertratanakul Y. Development of a shoulder pain and disability index. Arthritis Care Res. 1991;4(4):143-9.

2. Luime JJ, Koes BW, Hendriksen IJ, Burdorf A, Verhagen AP, Miedema HS, et al. Prevalence and incidence of shoulder pain in the general population; a systematic review. Scand J Rheumatol. 2004;33(2):73-81.

3. Membrilla-Mesa MD, Cuesta-Vargas AI, Pozuelo-Calvo R, Tejero-Fernández V, Martín-Martín L, Arroyo-Morales M. Shoulder pain and disability index: cross cultural validation and evaluation of psychometric properties of the Spanish version. Health and Quality of Life Outcomes. 2015;13(1):200.

4. Yao M, Yang L, Cao ZY, Cheng SD, Tian SL, Sun YL, et al. Translation and cross-cultural adaptation of the Shoulder Pain and Disability Index (SPADI) into Chinese. Clin Rheumatol. 2017;36(6):1419-26.

5. Phongamwong C, Choosakde A. Reliability and validity of the Thai version of the Shoulder Pain and Disability Index (Thai SPADI). Health and quality of life outcomes. 2015;13:136-.

6. Pribicevic. The epidemiology of shoulder pain: a narrative review of the literature. In: Ghosh S, editor. Pain in perspective. https://www.intechopen.com/books/pain-in-perspective/the-epidemiology-of-shoulder-pain-a-narrative-review-of-the-literature: IntechOpen; 2012.

7. Zainal Abidin N, Mohd. Rohani J, Nadia Nordin A, Md. Zein R, Satik anak Ayak A. Financial Impact and Causes of Chronic Musculoskeletal Disease Cases in Malaysia Based on Social Security Organization of Malaysia Claims Record. 2018. 2018;7(3.24):5.

8. Carvajal Bedoya G, Davis LA, Hirsh JM. Patient-Reported Outcomes in Rheumatology Patients With Limited English Proficiency and Limited Health Literacy. Arthritis Care & Research. 2020;72(S10):738-49.

9. Buchbinder R, Ramiro S, Huang H, Gagnier JJ, Jia Y, Whittle SL. Measures of Adult Shoulder Function. Arthritis Care & Research. 2020;72(S10):250-93.

10. Angst F, Schwyzer HK, Aeschlimann A, Simmen BR, Goldhahn J. Measures of adult shoulder function: Disabilities of the Arm, Shoulder, and Hand Questionnaire (DASH) and its short version (QuickDASH), Shoulder Pain and Disability Index (SPADI), American Shoulder and Elbow Surgeons (ASES) Society standardized shoulder assessment form, Constant (Murley) Score (CS), Simple Shoulder Test (SST), Oxford Shoulder Score (OSS), Shoulder Disability Questionnaire (SDQ), and Western Ontario Shoulder Instability Index (WOSI). Arthritis Care Res (Hoboken). 2011;63 Suppl 11:S174-88.

11. Schmidt S, Ferrer M, Gonzalez M, Gonzalez N, Valderas JM, Alonso J, et al. Evaluation of shoulder-specific patient-reported outcome measures: a systematic and standardized comparison of available evidence. J Shoulder Elbow Surg. 2014;23(3):434-44.

12. Devi. Development of Marathi version of cross culture adaptation, reliabilityh and validity of shoulder and disability index. International journal of science and research. 2015;6(7):5.

13. Martins J, Napoles BV, Hoffman CB, Oliveira AS. Versão Brasileira do Shoulder Pain and Disability Index: tradução, adaptação cultural e confiabilidade. Brazilian Journal of Physical Therapy. 2010;14:527-36.

14. Vrouva S, Batistaki C, Koutsioumpa E, Kostopoulos D, Stamoulis E, Kostopanagiotou G. The Greek version of Shoulder Pain and Disability Index (SPADI): translation, cultural adaptation, and validation in patients with rotator cuff tear. Journal of orthopaedics and traumatology : official journal of the Italian Society of Orthopaedics and Traumatology. 2016;17(4):315-26.

15. Marchese C, Cristalli G, Pichi B, Manciocco V, Mercante G, Pellini R, et al. Italian cross-cultural adaptation and validation of three different scales for the evaluation of shoulder pain and dysfunction after neck dissection: University of California - Los Angeles (UCLA) Shoulder Scale, Shoulder Pain and Disability Index (SPADI) and Simple Shoulder Test (SST). Acta Otorhinolaryngol Ital. 2012;32(1):12-7.

16. Yatheendra Kumar Gadam SS, Apparao Parchava, Siva C Kumar, Siva Jyothi Neerukonda, Neethui Kambarthi. Reliability and validity of the Indian (Telugu) version of the shoulder pain and disability index. Journal of clinical and diagnostic research. 2018;12(3):4.

17. Jeldi AJ, Aseer AL, Dhandapani AG, Roach KE. Cross-cultural adaption, reliability and validity of an Indian (Tamil) version for the Shoulder Pain and Disability Index. Hong Kong Physiotherapy Journal. 2012;30(2):99-104.

18. Angst F, Goldhahn J, Pap G, Mannion AF, Roach KE, Siebertz D, et al. Cross-cultural adaptation, reliability and validity of the German Shoulder Pain and Disability Index (SPADI). Rheumatology (Oxford). 2007;46(1):87-92.

19. Bumin G, Tüzün EH, Tonga E. The Shoulder Pain and Disability Index (SPADI): Cross-cultural adaptation, reliability, and validity of the Turkish version. Journal of Back and Musculoskeletal Rehabilitation. 2008;21:57-62.

20. Christiansen DH, Andersen JH, Haahr JP. Cross-cultural adaption and measurement properties of the Danish version of the Shoulder Pain and Disability Index. Clinical Rehabilitation. 2012;27(4):355-60.

21. Ebrahimzadeh MH, Birjandinejad A, Golhasani F, Moradi A, Vahedi E, Kachooei AR. Cross-cultural adaptation, validation, and reliability testing of the Shoulder Pain and Disability Index in the Persian population with shoulder problems. Int J Rehabil Res. 2015;38(1):84-7.

22. Jamnik H, Spevak MK. Shoulder Pain and Disability Index: validation of Slovene version. Int J Rehabil Res. 2008;31(4):337-41.

23. Thoomes-de Graaf M, Scholten-Peeters GG, Duijn E, Karel Y, Koes BW, Verhagen AP. The Dutch Shoulder Pain and Disability Index (SPADI): a reliability and validation study. Qual Life Res. 2015;24(6):1515-9.

24. Malaysia MoE. Malaysia Education Blueprint 2013-2015 (Preschool to Post Secondary Education): Kementerian Pendidikan Malaysia; 2013.

25. Deo GS. Malaysia Baharu 2019, Buku Rasmi Tahunan. Malaysia: Jabatan Penerangan Malaysia; 2020.

26. Gagnier JJ, Lai J, Mokkink LB, Terwee CB. COSMIN reporting guideline for studies on measurement properties of patient-reported outcome measures. Qual Life Res. 2021.

27. Beaton DE, Bombardier C, Guillemin F, Ferraz MB. Guidelines for the process of cross-cultural adaptation of self-report measures. Spine (Phila Pa 1976). 2000;25(24):3186-91.

28. Mokkink LB, Prinsen CA, Patrick DL, Alonso J, Bouter LM, de Vet HC, et al. COSMIN Study Design checklist for Patient-reported outcome measurement instruments. 2019.

29. Terwee CB, Bot SD, de Boer MR, van der Windt DA, Knol DL, Dekker J, et al. Quality criteria were proposed for measurement properties of health status questionnaires. J Clin Epidemiol. 2007;60(1):34-42.

30. Tsang S, Royse CF, Terkawi AS. Guidelines for developing, translating, and validating a questionnaire in perioperative and pain medicine. Saudi J Anaesth. 2017;11(Suppl 1):S80-S9.

31. Prinsen CA, Mokkink LB, Bouter LM, Alonso J, Patrick DL, De Vet HC, et al. COSMIN guideline for systematic reviews of patient-reported outcome measures. Quality of Life Research. 2018;27(5):1147-57.

32. Coltman T, Devinney TM, Midgley DF, Venaik S. Formative versus reflective measurement models: Two applications of formative measurement. Journal of Business Research. 2008;61(12):1250-62.

33. Haefeli M, Elfering A. Pain assessment. Eur Spine J. 2006;15 Suppl 1(Suppl 1):S17-24.

34. Guillemin F, Bombardier C, Beaton D. Cross-cultural adaptation of health-related quality of life measures: literature review and proposed guidelines. J Clin Epidemiol. 1993;46(12):1417-32.

35. Cynthia N. Measurement of joint motion: A guide to goniometry. Fifth Edition ed. Philadelphia: E. A Davis Company; 2016.

36. Harris PA, Taylor R, Thielke R, Payne J, Gonzalez N, Conde JG. Research electronic data capture (REDCap)—A metadata-driven methodology and workflow process for providing translational research informatics support. Journal of Biomedical Informatics. 2009;42(2):377-81.

37. Patridge EF, Bardyn TP. Research Electronic Data Capture (REDCap). J Med Libr Assoc. 2018;106(1):142-4.

38. Davis LL. Instrument review: Getting the most from a panel of experts. Applied Nursing Research. 1992;5(4):194-7.

39. Hair JF, Black WC, Babin BJ, Anderson RE. Multivariate Data Analysis: Pearson Education Limited; 2013.

40. Furtado R, Nazari G, MacDermid JC. A systematic review of the cross-cultural adaptations and measurement properties of the Shoulder Pain and Disability Index. Hand Therapy. 2019;24(4):107-15.

41. Bolarinwa O. Principles and methods of validity and reliability testing of questionnaires used in social and health science researches. Nigerian Postgraduate Medical Journal. 2015;22(4):195-201.

42. Koo TK, Li MY. A Guideline of Selecting and Reporting Intraclass Correlation Coefficients for Reliability Research. J Chiropr Med. 2016;15(2):155-63.

43. Dancey CP, Reidy J. Statistics Without Maths for Psychology: Pearson/Prentice Hall; 2007.

44. MacDermid JC, Solomon P, Prkachin K. The Shoulder Pain and Disability Index demonstrates factor, construct and longitudinal validity. BMC Musculoskelet Disord. 2006;7:12.

45. Roddey TS, Olson SL, Cook KF, Gartsman GM, Hanten W. Comparison of the University of California-Los Angeles Shoulder Scale and the Simple Shoulder Test with the shoulder pain and disability index: single-administration reliability and validity. Phys Ther. 2000;80(8):759-68.

46. Fritz CO, Morris PE, Richler JJ. Effect size estimates: current use, calculations, and interpretation. J Exp Psychol Gen. 2012;141(1):2-18.

S1_Fig

Click here to access/download
Supporting Information
S1_Fig, RedCap Online Demographics Form.docx

Click here to access/download

Supporting Information

S2_Fig, Malay Shoulder Pain and Disability Index (M-SPADI).docx

S3_Fig

Click here to access/download
Supporting Information
S3_Fig, Content Validity Form.docx
