# Supplementary material for: Cross-cultural adaptation and measurement properties of the Malay Shoulder Pain and Disability Index
Source: PLoS One. 2022 Mar 18;17(3):e0265198. doi: 10.1371/journal.pone.0265198 (PMC8932568; doi:10.1371/journal.pone.0265198)
Supplement: S3 Appendix — (DOCX) [file pone.0265198.s003.docx]

[S3 Appendix.](C:\\Users\\Jozald\\AppData\\Local\\Temp\\S4 Fig. Content Validity Form.pdf) Content Validity Form

CONTENT VALIDITY FORM

INDEKS KESAKITAN DAN KETIDAKUPAYAAN BAHU

NAME:

DESIGNATION:

Dear Professor/ Doctor/ Sir/Madam,

**Directions**: This tool asks for your evaluation of the questionnaire “INDEKS KESAKITAN DAN KETIDAKUPAYAAN BAHU” to establish its validity. You are requested to rate each question with reference to the questionnaire attached. Please feel free to relay your comments/ suggestions/corrections regarding this questionnaire if applicable.

Rating scale: 1=strongly disagree	2=disagree 3= agree 4= strongly agree

Domain 1: Skala Kesakitan

| Question | This question is consistent with the questionnaire and domain explored. | This question is representative of the concepts explored by the questionnaire. | This question is relevant to the concepts explored by the questionnaire. | The wordings in the question are clear and easily understood. | comments |
| --- | --- | --- | --- | --- | --- |
| Q1 | ①②③④ | ①②③④ | ①②③④ | ①②③④ |  |
| Q2 | ①②③④ | ①②③④ | ①②③④ | ①②③④ |  |
| Q3 | ①②③④ | ①②③④ | ①②③④ | ①②③④ |  |
| Q4 | ①②③④ | ①②③④ | ①②③④ | ①②③④ |  |
| Q5 | ①②③④ | ①②③④ | ①②③④ | ①②③④ |  |

Domain 2: Skala Ketidakupayaan

| Question | This question is consistent with the questionnaire and domain explored. | This question is representative of the concepts explored by the questionnaire. | This question is relevant to the concepts explored by the questionnaire. | The wordings in the question are clear and easily understood. | comments |
| --- | --- | --- | --- | --- | --- |
| Q6 | ①②③④ | ①②③④ | ①②③④ | ①②③④ |  |
| Q7 | ①②③④ | ①②③④ | ①②③④ | ①②③④ |  |
| Q8 | ①②③④ | ①②③④ | ①②③④ | ①②③④ |  |
| Q9 | ①②③④ | ①②③④ | ①②③④ | ①②③④ |  |
| Q10 | ①②③④ | ①②③④ | ①②③④ | ①②③④ |  |
| Q11 | ①②③④ | ①②③④ | ①②③④ | ①②③④ |  |
| Q12 | ①②③④ | ①②③④ | ①②③④ | ①②③④ |  |
| Q13 | ①②③④ | ①②③④ | ①②③④ | ①②③④ |  |
